# Phytochemical Profiling Studies of Alkaloids and Coumarins from the Australian Plant *Geijera parviflora* Lindl. (*Rutaceae*) and Their Anthelmintic and Antimicrobial Assessment

**DOI:** 10.3390/metabo14050259

**Published:** 2024-04-30

**Authors:** Deepika Dugan, Rachael J. Bell, Robert Brkljača, Colin Rix, Aya C. Taki, Robin B. Gasser, Sylvia Urban

**Affiliations:** 1 Marine and Terrestrial Natural Product (MATNAP) Research Group, School of Science (Applied Chemistry and Environmental Science), RMIT University, Melbourne, VIC 3001, Australia; s3646443@student.rmit.edu.au (D.D.); rbell0030@gmail.com (R.J.B.); colin.rix@rmit.edu.au (C.R.); 2 Monash Biomedical Imaging, Monash University, Clayton, VIC 3168, Australia; robert.brkljaca@monash.edu; 3 Department of Veterinary Biosciences, Faculty of Science, Melbourne Veterinary School, University of Melbourne, Parkville, VIC 3010, Australia; aya.taki@unimelb.edu.au (A.C.T.); robinbg@unimelb.edu.au (R.B.G.)

**Keywords:** alkaloids, anthelmintics, auraptene, coumarins, flindersine, *Geijera parviflora*, geiparvarin, *Haemonchus contortus*, phytochemical profiling

## Abstract

Phytochemical profiling followed by antimicrobial and anthelmintic activity evaluation of the Australian plant *Geijera parviflora,* known for its customary use in Indigenous Australian ceremonies and bush medicine, was performed. In the present study, seven previously reported compounds were isolated including auraptene, 6′-dehydromarmin, geiparvarin, marmin acetonide, flindersine, and two flindersine derivatives from the bark and leaves, together with a new compound, chlorogeiparvarin, formed as an artefact during the isolation procedure and isolated as a mixture with geiparvarin. Chemical profiling allowed for a qualitative and quantitative comparison of the compounds in the leaves, bark, flowers, and fruit of this plant. Subsequently, a subset of these compounds as well as crude extracts from the plant were evaluated for their antimicrobial and anthelmintic activities. Anthelmintic activity assays showed that two of the isolated compounds, auraptene and flindersine, as well as the dichloromethane and methanol crude extracts of *G. parviflora*, displayed significant activity against a parasitic nematode (*Haemonchus contortus*). This is the first report of the anthelmintic activity associated with these compounds and indicates the importance of such fundamental explorations for the discovery of bioactive phytochemicals for therapeutic application(s).

## 1. Introduction

*Geijera parviflora* Lindl. belongs to the plant genus *Geijera* Schott of the family *Rutaceae* Juss. (rue and citrus), which consists of six species that are all native to Oceania. *G. parviflora* is one of the three *Geijera* species that are endemic to Australia. Commonly known as Wilga, *G. parviflora* is a small tree that grows to 8 m, and it is endemic to inland areas of Eastern Australia. *G. parviflora* leaves and bark are used customarily for Indigenous Australian ceremonial purposes and bush medicine [1]. The species is hardy and drought tolerant, and has been used as sheep fodder by Australian farmers during times of drought [2].

**Figure 1:**
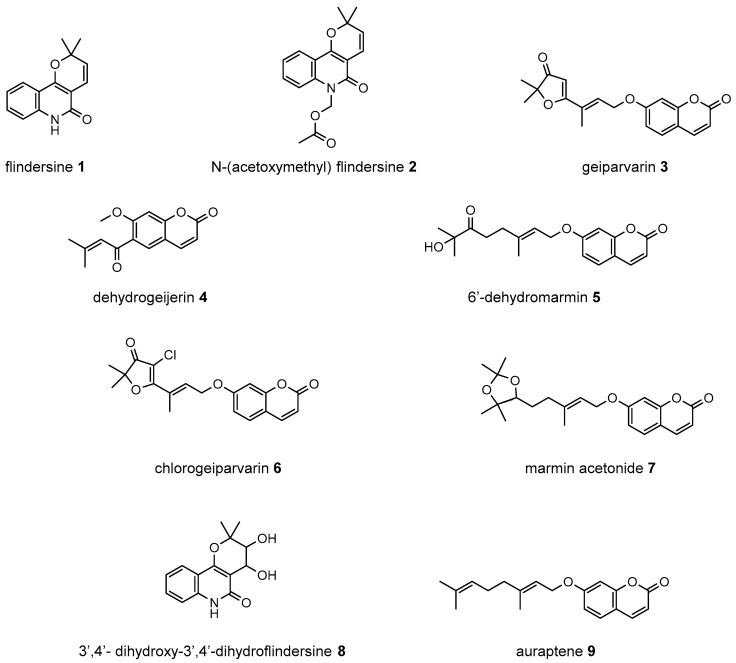
Compounds from the bark and leaves of *Geijera parviflora*.

In customary usage, *G. parviflora* leaves were utilised to treat pain, fresh leaves were chewed to relieve toothache, and dried leaves were powdered and smoked with other plant material to induce intoxication or drowsiness [3]. While the constituents responsible for the analgesic properties reported in the traditional use of this plant have yet to be confirmed, several isolated coumarins and alkaloids from *G. parviflora* have anti-inflammatory activity, and it has been proposed that the alkaloid flindersine **1** and its derivative N-(acetoxymethyl) flindersine **2** (Figure 1) may contribute to this activity through the inhibition of prostaglandin E2, an important mediator of inflammation [4].

It has also been observed that the leaves of *G. parviflora* exhibit selective palatability as fodder for sheep, whereby some morphologically similar plants are readily consumed by sheep while others are not [5]. Two coumarins, geiparvarin **3** and dehydrogeijerin **4** (Figure 1), were isolated by Lahey and Macleod from specimens deemed either ‘readily eaten’ or ‘unpalatable’ [5]. It was found that dehydrogeijerin **4** was only present in the unpalatable variety, and that geiparvarin **3** was only in the readily eaten variety [5]. Coumarins **3** and **4** display significant anticancer and anti-inflammatory activities, respectively [6,7]. Geiparvarin **3** is a major compound isolated from *G. parviflora*, and both **3** and its related analogues display cytotoxic, cytostatic, and selective anti-tumour activity [8].

In a closely related species, *G. salicifolia* (Scrub Wilga, Greenheart, Green Satinheart), which is a long-lived, drought-tolerant, and hardy plant (also commonly called Wilga), the leaves have a similar customary use to *G. parviflora*. According to the Dharawal pharmacopeia collection recorded by Auntie Frances Bodkin, a Dharawal elder of the Dharawal people, *G. salicifolia* is used for pain relief, whereby the leaves are chewed to alleviate toothache, and the vapours from the hot leaves are used to relieve headache [9].

Dominant classes of natural products previously isolated from *G. parviflora* include coumarins, alkaloids, terpenes, terpenoids, and phenolics. These compounds have displayed analgesic, anthelmintic, anti-inflammatory, anticancer, and antimicrobial effects amongst other activities, e.g., monoamine oxidase inhibition (geiparvarin **3)**, collagen III suppression (N-(acetoxymethyl) flindersine **2**), and antifungal activity (auraptene **9**), and, thus, warrant further investigation [4,10,11,12,13,14].

Previous studies have mostly characterised the constituents of the essential oils from the leaves of this plant, with fewer studies on the other plant parts, such as the fruit, bark, and leaf extracts, and no studies of the flowers. In the present work, large-scale isolation of *G. parviflora* coumarins and alkaloids from the bark and leaves was performed, so that some of its major constituent compounds could be evaluated for other pharmacological activities, such as anthelmintic activity and antimicrobial activity, in addition to their known activities which have been recently reviewed by the authors in a separate publication [15].

This study reports the first chemical profiling comparison of the flowers, leaves, bark, and fruit of *G. parviflora* and is the first report of anthelmintic activity of the plant extract and some of its constituents against an economically important parasitic nematode, called *Haemonchus contortus* (the barber’s pole worm) [16]. This parasitic nematode is a gastrointestinal pathogen that affects small ruminants including sheep and goats. Specifically, the dichloromethane and methanol extracts of the bark, flowers, and leaves, as well as five isolated compounds from the leaves of *G. parviflora* were tested for anthelmintic activity against *H. contortus.* This parasite represents a larger group of nematodes (order *Strongylida*) that affect a wide range of animals including humans [17].

Additionally, two major coumarins isolated from the bark of *G. parviflora*, geiparvarin **3** and 6′- dehydromarmin **5**, were tested for their antimicrobial activity against the five key pathogens, *E. coli, S. aureus* (MRSA), *K. pneumoniae, A. baumannii, P. aeruginosa* (designated “ESKAP”), as well as the fungi *C. neoformans* and *C. albicans*.

## 2. Materials and Methods

### 2.1. Chemicals and Reagents

Experimental procedures were conducted using analytical grade (AR), HPLC grade and LC-MS hypergrade organic solvents, deuterated solvents, and Milli-Q water.

### 2.2. Plant Material

A specimen identified and provided by the curator at the Royal Botanic Gardens Cranbourne, Victoria, Australia, was assigned voucher code 2019_05. It was then separated into the leaves (2019_05a) and bark (2019_05b) components of the plant, respectively. Additional specimens were identified and provided by the curator at Maranoa Botanic Gardens Balwyn, Victoria, Australia, which were assigned voucher codes 2021_19, 2022_05, and 2022_09. The specimens were then separated into the leaves (2021_19a and 2022_09a), flowers (2022_05a), bark (2021_19b and 2022_09b), and fruit (2021_19c and 2022_09c) components of the plant, respectively. The specimens were stored at −80 °C until extractions were conducted.

### 2.3. Extraction and Analysis

Specimens of the flowers, leaves, bark, and fruit were pulverised and then subjected to sequential solvent extraction (trituration) with dichloromethane (DCM) and then methanol (MeOH), respectively, to yield corresponding non-polar and polar crude extracts. These were concentrated under reduced pressure and then dried at 35 °C under a stream of nitrogen. The dried crude extracts were then dissolved in LC-MS hypergrade MeOH for analysis.

Extractions and HPLC-DAD/LC-MS comparisons of the phytochemical profiles of the crude extracts were conducted on 2.5 g specimens of the four different plant parts (specimen voucher codes: 2022_05a (flowers); 2022_09a (leaves), 2022_09b (bark), and 2022_09c (fruit)). The crude extracts were dissolved in LC-MS hypergrade MeOH and analysed at a concentration of 2 mg/mL.

DCM and MeOH crude extracts were obtained from 10 g specimens of the leaves, bark, and flowers (specimen voucher codes 2021_19a, 2021_19b, and 2022_05a, respectively) and were subjected to anthelmintic activity assays. The MeOH crude extracts from the bark and leaves both naturally separated into solid and resinous components during rotary evaporation, yielding solid and resinous crude extracts which were all assessed for their anthelmintic activity along with the DCM crude extracts. The fruits and flowers were not further evaluated for anthelmintic activity due to the limited amounts available.

Larger-scale extractions were conducted from the remaining plant material consisting of a 50 g specimen of the bark (specimen voucher code 2019_05b) and 250 g specimen of the leaves (specimen voucher code 2021_19a) to yield DCM and MeOH crude extracts. The DCM crude extracts obtained from these specimens were prioritised for subsequent fractionation and compound isolation (see extraction Schemes S1.1 and S2.1 in Supplementary Materials S1 and S2). The DCM crude extracts were fractionated using flash silica column chromatography. Crude extracts and fractions were analysed using HPLC-DAD, and selected fractions were analysed using LC-MS. Purified compounds were subsequently isolated via semi-preparative HPLC, and their structures were confirmed with HPLC-DAD, LC-MS, and NMR spectroscopic analyses.

### 2.4. Silica Flash Column Chromatography

Silica flash column chromatography was performed using ~40–50 g Merck Silica gel 60 (0.040–0.063 mm) with a 1:10 loading capacity and 20% stepwise elution gradient operating with increasingly polar solvents—100% n-hexane to 100% DCM to 100% EtOAc and finally to 100% MeOH (see extraction Schemes S1.1 and S2.1 in Supplementary Materials S1 and S2).

### 2.5. HPLC-DAD Analysis

Reversed phase HPLC analysis was conducted using a Dionex P680 solvent delivery system, fitted with a 250 × 4.6 mm Agilent ZORBAX Eclipse Plus (5 μm) C18 column (Agilent, Santa Clara, CA, USA). Injection volumes of 10 μL were delivered via an ASI-100 automated sample injector with a flow rate of 1 mL/min. UV profiles between 190 and 600 nm were obtained with a PDA-100 photodiode array detector. The HPLC instrument modules were operated using “Chromeleon” software version 6.8 SR16 (Build 5387). HPLC analysis was conducted using a standard gradient method of 0–2 min 10% CH_3_CN: 90% H_2_O; 14–24 min 75% CH_3_CN: 25% H_2_O; 26–30 min 100% CH_3_CN; and 32–40 min 10% CH_3_CN: 90% H_2_O. Various isocratic methods were tested on selected fractions for the isolation of compounds with semi-preparative HPLC.

### 2.6. Semi-Preparative HPLC Isolation

Reversed phase semi-preparative HPLC was carried out on 30–50 μL injections on a Varian Prostar 210 solvent delivery system equipped with a 250 × 9.4 mm, Agilent Eclipse XDB C18 (5 μm) column (Agilent, Santa Clara, CA, USA) and a Prostar 335 PDA detector operated using “Star Workstation” software version 6.30 (Build 5); monitoring at selected wavelengths (210 and 330 nm). A range of isocratic HPLC purification methods were used to isolate compounds from the bark and leaves and these ranged from solvent compositions between 40 and 80% CH_3_CN: 60 and 20% H_2_O and flow rates of 3.5–4.0 mL/min (for specific details of these isolations see extraction Schemes S1.1 and S2.1 in Supplementary Materials S1 and S2).

### 2.7. LC-MS Analysis

LC-MS analysis of pure compounds isolated from the bark of *G. parviflora* was conducted in both the positive and negative electrospray ionisation modes with a capillary voltage of 4.5 kV. The LC system was equipped with an Agilent 1100 series solvent delivery module fitted with a 150 × 4.6 mm Agilent ZORBAX Eclipse Plus (5 μm) C18 column (Agilent, Santa Clara, CA, USA) using 50% CH_3_CN/H_2_O at a flow rate of 0.2 mL/min, and a 100 series autosampler, column switcher, and UV detector. The LC system was controlled using “Analyst” software version 1.6.3. The MS module was an Applied Biosystems MDS Sciex Q Trap LC/MS/MS system.

LC-MS analysis of crude extracts from various plant parts as well as pure compounds isolated from the leaves of *G. parviflora* was conducted in both positive and negative electrospray ionisation modes with a capillary voltage of 4 kV. The LC system was equipped with an Agilent 1260 Infinity II quaternary pump solvent delivery module fitted with a 250 × 4.6 mm Agilent ZORBAX Eclipse Plus (5 μm) C18 column (Agilent, Santa Clara, CA, USA), a 1260 Infinity II Multisampler, and 1260 Infinity II Diode Array Detector HS. Analysis was carried out using a standard gradient method of 0–2 min 10% CH_3_CN: 90% H_2_O; 14–24 min 75% CH_3_CN: 25% H_2_O; 26–30 min 100% CH_3_CN; and 32–40 min 10% CH_3_CN: 90% H_2_O at a flow rate of 1 mL/min. The LC system was controlled using “OpenLab CDS” software version 2.6.0.691. The MS module was an Agilent InfintyLab single quadrupole LC/MSD system.

### 2.8. NMR Spectroscopy

^1^H (500 MHz) and ^13^C (125 MHz) spectra were acquired in CDCl_3_ or CD_3_OD, on a 500 MHz Agilent DD2 NMR spectrometer operated using VnmrJ software version 4.2 rev. A, with reference to solvent signals (δ 7.26 ppm and 77.0 ppm for CDCl_3_, or δ 3.31 ppm and 49.0 ppm for CD_3_OD). Two-dimensional NMR spectra were recorded on the same instrument, and these included gCOSY, HSQCAD, and gHMBCAD NMR experiments. A Bruker 300 MHz Avance III NMR spectrometer operated with Bruker TopSpin software version 3.6.5, with reference to solvent signals (δ 7.26 ppm and 77.0 ppm for CDCl_3_ or δ 3.31 ppm and 49.0 ppm for CD_3_OD) was also used for the acquisition of rapid ^1^H NMR experiments using a Bruker SampleCase^TM^ 24-slot autosampler. “Bruker TopSpin”, “MestReNova” and “ACD Spectrus” software was used for processing the NMR data.

### 2.9. Compound Characterisation

flindersine **1** (white powder), 16 mg (0.09% of dry weight of bark specimen), 2.4 mg (0.003% of dry weight of leaves specimen), [M + H]^+^ *m*/*z* 228, [2M + H]^+^ *m*/*z* 455, [2M + Na]^+^ *m*/*z* 477 and [3M + Na]^+^ *m*/*z* 704 was identified based on its NMR data and its molecular mass which were in accordance with literature values for this compound [4].

N-(acetoxymethyl) flindersine **2** (white powder), 1.3 mg (0.007% of dry weight of bark specimen), 3.1 mg (0.004% of dry weight of leaves specimen), [M + H]^+^ *m*/*z* 300, [2M + Na]^+^ *m*/*z* 621 and [M-C_2_H_3_O_2_ (acetoxy)]^+^ *m*/*z* 240 was identified based on its NMR data and its molecular mass which were in accordance with literature values for this compound [4].

geiparvarin **3** (white powder), 4.2 mg (0.02% of dry weight of bark specimen), 1.3 mg (0.002% of dry weight of leaves specimen), [M + H]^+^ *m*/*z* 327 and [2M + H]^+^ *m*/*z* 653; [M − H]^−^ *m*/*z* 325 was identified based on its NMR data and molecular mass which were in accordance with the literature values for this compound [5].

6′-dehydromarmin **5** (white powder) 1.8 mg (0.01% of dry weight of bark specimen), 1.7 mg (0.003% of dry weight of leaves specimen), [M + H]^+^ *m*/*z* 331 and [2M + Na]^+^ *m*/*z* 683; [M + Cl]^−^ *m*/*z* 365 was identified based on its NMR data and its molecular mass which were in accordance with literature values [18].

chlorogeiparvarin **6** (white powder) 0.2 mg (0.001% of dry weight of bark specimen), isolated as a 1:1.2 mixture with geiparvarin **3**. Both the [M + Na]^+^ ions for geiparvarin **3** and chlorogeiparvarin **6** at *m*/*z* 349 and 383, respectively, were detected in the LC-MS. ^1^H NMR (500 MHz, CDCl_3_) ^13^C NMR (125 MHz, CDCl_3_); see Table 1 (NMR spectra are provided in Supplementary Materials S3 (Figure S3.1)).

marmin acetonide **7** (white powder) 0.7 mg (0.004% of dry weight of bark specimen), LC-MS [M-OH]^+^ *m*/*z* 355 and 297 [M-C_3_H_7_O_2_]^+^ was identified based on its NMR data and its molecular mass which were in accordance with literature values for this compound [18].

3′,4′-dihydroxy-3′,4′-dihydroflindersine **8** (white powder) 0.1 mg (0.0006% of dry weight of bark specimen), LC-MS [M + H]^+^ *m*/*z* 262, was identified based on its NMR data and molecular mass which were in accordance with literature values for this compound [19].

auraptene **9** (white powder) 2.8 mg (0.02% of dry weight of leaves specimen), LC-MS [M + H]^+^ *m*/*z* 299, [2M + H]^+^ *m*/*z* 597 and [2M + Na]^+^ *m*/*z* 619, was identified based on its NMR data and molecular mass which were in accordance with literature values for this compound [17].

### 2.10. Anthelmintic Activity Assessment

The *G. parviflora* DCM and MeOH crude extracts and the purified compounds **1**, **2**, **3**, **5**, and **9** were evaluated for activity against exsheathed third-stage larvae (xL3s) of the *H. contortus* (Haecon-5 strain) nematode worm, to evaluate their effects on larval motility and/or development using an established protocol [20]. The assessment of anthelmintic activity was carried out in a screening (extracts) and a dose-response assay (compounds) using for exsheathed third-stage larvae (xL3s) of *Haemonchus contortus* (Haecon-5 strain). *H. contortus* were maintained in experimental sheep and procured in accordance with the institutional animal ethics guidelines and the regulations of Australia (permit no. 23983; University of Melbourne) [20]. Immediately prior to use, third-stage larvae (L3s) were exsheathed and sterilised by incubation in 0.15% (*v*/*v*) bleach at 38 °C for 20 min, followed by washes in sterile saline at room temperature (22–24 °C). After the last wash, xL3s were suspended in sterile lysogeny broth (LB) supplemented with 100 IU/mL of penicillin, 100 μg/mL of streptomycin, and 0.25 μg/mL of amphotericin B (Fungizone^®^, Thermo Fisher Scientific, Waltham, MA, USA)—designated LB* [20]. *G. parviflora* extracts were prepared at a concentration of 1 mg/mL (in 50 µL of LB*; final assay concentration of 0.5 mg/mL) and compounds were prepared in two-fold serial dilution, starting at a concentration of 100 μM (18-points; in 50 μL of LB*; final assay concentrations of 50 μM to 0.76 nM), in 96-well plates (cat. no. 3596; Corning, USA) with larvae dispensed in 50 μL at a density of 300 [20]. LB* + 0.5% DMSO serving as negative control and two commercial anthelmintic compounds, monepantel (Zolvix™; Elanco, Australia) and moxidectin (Cydectin^®^; Virbac, France), were prepared as positive controls and applied to the 96-well microtiter plates in the same manner (Corning, USA). Following a 168-h incubation at 38 °C, 10% CO_2_ with >90% humidity, worm activity was captured using a WmicroTracker ONE (Phylumtech, Sunchales, Santa Fe, Argentina). Over a period of 15 min, interference of an infrared beam in individual wells was recorded as a worm ‘activity count’. Activity counts were then normalised to the positive and negative controls using the program Prism (v.9.1.0 GraphPad Software, San Diego, CA, USA) to remove plate-to-plate variation. An extract was deemed as having activity if it reduced xL3s motility by ≥70% and/or inhibited larval development after 168 h of incubation. To observe compound effects, the half-maximal inhibitory concentrations (IC_50_ values) were estimated. Worms were fixed with 40 µL of Lugol’s solution (Sigma-Aldrich, St. Louis, MO, USA), and assessed microscopically via the development of a mouth. Additionally, a compound that induced a non-wildtype phenotype (visible microscopically at 200-times magnification) was recorded.

Individual crude extracts were evaluated at a single concentration of 0.5 mg/mL (vehicle: DMSO), and the activity of the purified compounds was assessed in dose-response assays (50 µM to 0.2 µM; vehicle: DMSO). The compounds assessed were selected based on availability and chemical stability.

### 2.11. Antimicrobial Activity Assessment

Compounds **3** and **5** were submitted to Community for Open Antimicrobial Drug Discovery (CO-ADD). These compounds were selected based on availability and chemical stability and were evaluated in duplicate (n = 2) against seven microorganisms (five bacteria and two fungi) at a concentration of 32 µg/mL in a 384-well, non-binding surface plate (NBS) for each bacterial/fungal strain, keeping the final DMSO concentration to a maximum of 1% DMSO. All bacteria were cultured in cation-adjusted Mueller-Hinton broth (CAMHB) at 37 °C overnight. A sample of each culture was then diluted 40-fold in fresh broth and incubated at 37 °C for 1.5–3 h. The resultant mid-log phase cultures were diluted (CFU/mL measured by OD600), then added to each well of the compound containing plates, giving a cell density of 5 × 10^5^ CFU/mL and a total volume of 50 µL. All the plates were covered and incubated at 37 °C for 18 h without shaking. Fungal strains were cultured for 3 days on Yeast Extract-Peptone Dextrose (YPD) agar at 30 °C. A yeast suspension of 1 × 10^6^ to 5 × 10^6^ CFU/mL (as determined by OD530) was prepared from five colonies. The suspension was subsequently diluted and added to each well of the compound-containing plates giving a final cell density of fungi suspension of 2.5 × 10^3^ CFU/mL and a total volume of 50 µL. All plates were covered and incubated at 35 °C for 24 h without shaking. Inhibition of bacterial growth was determined measuring absorbance at 600 nm (OD600) using a Tecan M1000 Pro monochromator plate reader. Growth inhibition of *C. albicans* was determined measuring absorbance at 530 nm (OD530), while the growth inhibition of *C. neoformans* was determined measuring the difference in absorbance between 600 and 570 nm (OD600-570), after the addition of resazurin (0.001% final concentration) and incubation at 35 °C for an additional 2 h. The absorbance was measured using a Biotek Synergy HTX plate reader. The percentage of growth inhibition was calculated for each well, using negative control (medium only) and positive control (bacteria without inhibitors) on the same plate as the references. The significance of inhibition values was determined by modified Z-scores, calculated using the median and MAD of the samples (no controls) on the same plate. Samples with an inhibition value above 80% and Z-score above 2.5 for either replicate (n = 2 on different plates) were classed as actives. Colistin and vancomycin were used as positive bacterial inhibitor standards for Gram-negative and Gram-positive bacteria, respectively. Fluconazole was used as a positive fungal inhibitor standard for *C. albicans* and *C. neoformans*. The antibiotics were provided in four concentrations, with two above and two below its MIC value, and plated into the first eight wells of column 23 of the 384-well NBS plates. The quality control (QC) of the assays was determined by the antimicrobial controls and the Z’-factor (using positive and negative controls). Each plate was deemed to fulfil the quality criteria (pass QC), if the Z’-factor was above 0.4, and the antimicrobial standards showed a full range of activity, with full growth inhibition at their highest concentration, and no growth inhibition at their lowest concentration. The seven test microorganisms were *Staphylococcus aureus* MRSA (ATCC 43300), *Escherichia coli* (ATCC 25922), *Klebsiella pneumoniae* (ATCC 700603), *Acinetobacter baumanii* (ATCC19606), *Pseudomonas aeruginosa* (ATCC 27853), *Candida albicans* (ATCC 90028), and *Cryptococcus neoformans* var. grubii (H99; ATCC 208821).

## 3. Results and Discussion

A total of eight compounds were isolated in this study from both the bark and the leaves of *G. parviflora* (Figure 1). Compounds isolated from the bark included one new compound which was a chlorinated artefact assigned the name chlorogeiparvarin **6**, isolated as a mixture with geiparvarin; two previously described coumarins including geiparvarin **3** and 6′-dehydromarmin **5**; the alkaloids flindersine **1**, N-(acetoxymethyl) flindersine **2** and 3′,4′- dihydroxy-3′,4′-dihydroflindersine **8**; and one previously described artefact of isolation, marmin acetonide **7**. Compounds isolated from the leaves included **1**, **2**, **4**, **5**, and auraptene **9**. These compounds were represented by major peaks in the analytical HPLC chromatograms of the crude extracts and their constituent fractions (see Section 3.3).

### 3.1. Isolation of Compounds from the Bark of G. parviflora

Pulverised bark (50 g) from the specimen assigned voucher code 2019_05 yielded crude DCM and MeOH extracts after sequential solvent extraction (trituration). The DCM crude extract was prioritised for further fractionation based on its analytical HPLC profile which displayed a variety of chromophores of interest. Fractionation of the DCM crude extract of the bark on a silica column resulted in a total of 18 fractions. Seven compounds were isolated from two major fractions. The two major fractions prioritised for further isolation were specifically those which possessed chromophores with UV maxima between 300 and 350 nm, corresponding to coumarins as well as to pyranoquinolinone alkaloids (such as flindersine **1** and its derivatives), i.e., the two compound classes that are known to contribute to the pharmacological activity of *G. parviflora*.

The first two compounds isolated were the previously described coumarins 6′-dehydromarmin **5** (white powder, M + H]^+^ *m*/*z* 331 and [2M + Na]^+^ *m*/*z* 683; [M + Cl]^−^ *m*/*z* 365, inferring a molecular mass of 330 amu), and geiparvarin **3** (white powder, [M + H]^+^ *m*/*z* 327 and [2M + H]^+^ *m*/*z* 653; [M − H]^−^ *m*/*z* 325, inferring a molecular mass of 326 amu). 6′-dehydromarmin **5** displays anti-inflammatory and cytotoxic activities, whilst geiparvarin **3** is a monoamine oxidase B inhibitor and it also displays anticancer activity [4,21,22]. As both compounds had not been previously assessed for their antimicrobial activity against the ESKAP pathogens, they were further evaluated in antimicrobial assays at CO-ADD.

A subsequent compound, isolated via semi-preparative HPLC, representing a new structural derivative, was assigned as chlorogeiparvarin **6** (isolated as a 1:1.2 mixture with geiparvarin **3** based on the integration for the signals at δ 4.86 and 4.83 ppm, see Supplementary Materials S3 (Figures S3.2–S3.4). It is suspected that this chlorinated artefact **6** (Figure 1), is a product of the extraction and isolation procedure since the fraction was dissolved in chlorinated solvents such as dichloromethane and deuterated chloroform. The tabulated NMR data for this compound is provided in Table 1.

The structure was elucidated on the basis of a comparison of the ^1^H and ^13^C NMR chemical shifts with other compounds isolated (**3**, **5**) in this study and by comparison with the literature NMR data reported for geiparvarin **3** [5]. The NMR data, as expected, was very similar for the two compounds, being identical in the aromatic right-hand portion of the molecule and with chemical shift differences in the left-hand side which were consistent with the additional substituent at the 3” position. The presence of the halogen substituent was suggested due to the lack of a proton signal corresponding to position 3” in the ^1^H NMR spectrum (see Table 1 and Table 2), together with the LC-MS showing both the [M + Na]^+^ ions for geiparvarin **3** and chlorogeiparvarin **6** at *m*/*z* 349 and 383, respectively. The mixture containing chlorogeiparvarin **6** was unstable, degrading quickly, hence preventing any further characterisation (NMR spectra are provided in Supplementary Materials S3). Chlorinated artefacts are common in natural products and may form in solution due to the increased instability of the halogenated solvent from interactions with molecules and contaminants present in solution with chlorinated solvents [23].

Further compounds purified from the major fractions included:

N-(acetoxymethyl) flindersine **2**, which has been observed to display anti-inflammatory and collagen III suppression activities, both of which indicate its therapeutic potential to assist with pain relief and wound healing [4].

Marmin acetonide **7**, which was previously identified as the acetone ketal of marmin by Dreyer and Lee, is considered an artefact of the isolation procedure, caused by exposure to acetone [18]. In this current study, exposure to small amounts of acetone could have occurred since acetone was used to rinse all glassware prior to use. In the positive mode LR_MS, there was a peak at *m*/*z* 297 amu which corresponds to the loss of 75 amu from marmin acetonide which has a molecular mass of 372 amu. This could indicate the loss of C_3_H_7_O_2_ from the core structure of marmin acetonide. Whilst compound **7** has been previously described, its activity remains unknown [18].

Flindersine **1**, which has been isolated from many different species and genera across the *Rutaceae* family. It displays antibacterial, anti-inflammatory, collagen III suppression, and antifungal activities [4,13,24].

3′,4′-dihydroxy-3′,4′-dihydroflindersine **8**, which was previously isolated from *G. balansae,* a plant of the *Geijera* genus that is endemic to New Caledonia [19]. This is the first instance of **8** being reported from *G. parviflora*.

Due to insufficient quantities of some of the compounds obtained from the bark, further biological testing and evaluation was not carried out.

### 3.2. Isolation of Compounds from the Leaves of G. parviflora

Pulverised leaves (250 g) from the specimen assigned voucher code 2021_19 underwent sequential solvent extraction (trituration) with DCM and MeOH to yield crude extracts. The DCM crude extract was prioritised for further separation, and was fractionated on a silica column, resulting in a total of 34 fractions. Following analytical HPLC and low-resolution LC-MS analysis of selected fractions, a total of five compounds were purified via semi-preparative HPLC from three major fractions. These five compounds were confirmed to be the previously reported coumarins auraptene **9**, 6′-dehydromarmin **5**, geiparvarin **3**, as well as the alkaloids N-(acetoxymethyl) flindersine **2** and flindersine **1**, based on their NMR chemical shifts and molecular masses obtained via LC-MS which were all in accordance with the literature data [4,5,18,25].

The activities of all the compounds except auraptene **9**, have already been described in the previous section on the extraction of the bark. Auraptene **9**, also known as 7-geranyloxycoumarin is amongst the most abundant naturally occurring prenyloxy umbelliferone derivatives present in several genera of the *Rutaceae* and *Apiaceae* plant families [12]. It displays numerous activities including increase of collagen I expression, antibacterial, antifungal, antileishmanial, antidiabetic, anticancer, neuroprotective, and antioxidant activity [12,13,14,26].

### 3.3. Comparison of Phytochemical Profiles of the Flowers, Leaves, Bark, and Fruits of G. parviflora

**Figure 2:**
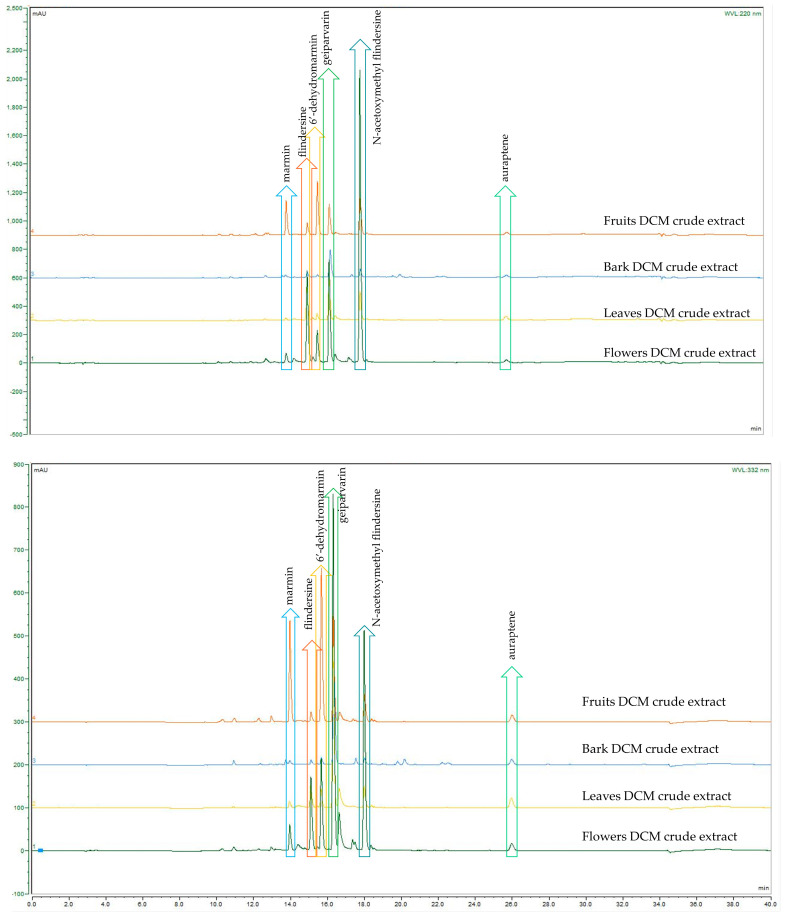
HPLC-DAD comparison of *G. parviflora* DCM extracts at 220 nm (**top**) and 332 nm (**bottom**).

**Figure 3:**
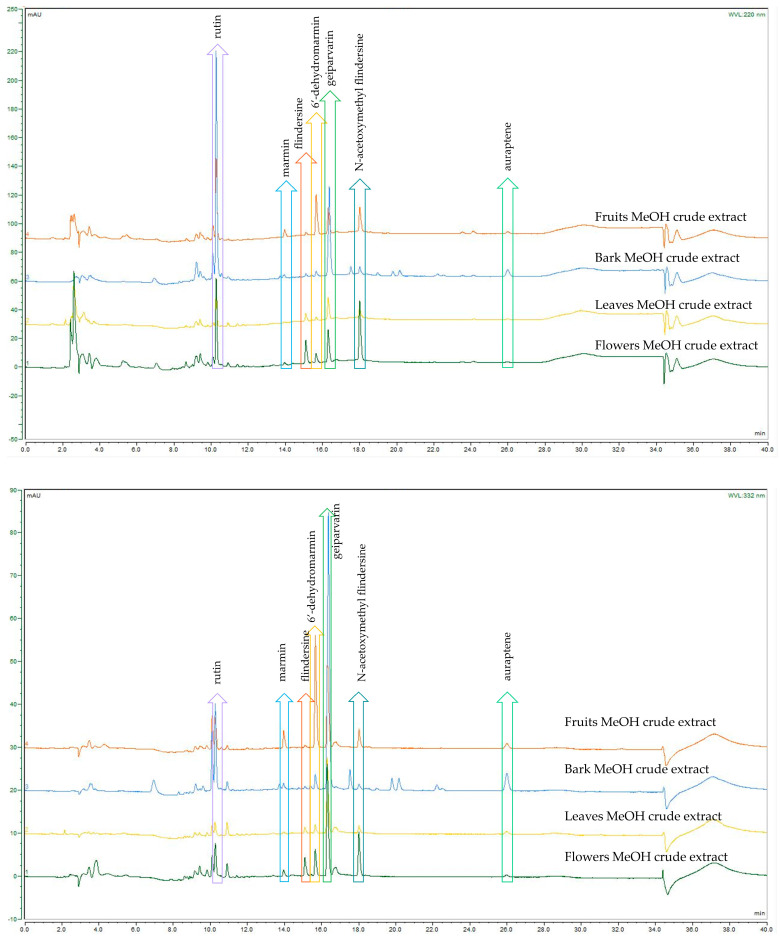
HPLC-DAD comparison of *G. parviflora* MeOH extracts at 220 nm (**top**) and 332 nm (**bottom**).

A comparison of the analytical HPLC chromatographic profiles of the crude DCM and MeOH extracts (all analysed at a concentration of 2 mg/mL) was conducted. These extracts were obtained from the four different plant parts of *G. parviflora*, namely the flowers, leaves, bark, and fruits. The motivation was to conduct a comparison of these extracts to observe which common metabolites were present, which were dominant, and if any other metabolites were present. The analysis permitted similar and dominant metabolites such as the coumarins, alkaloids, and the glycoside flavonol, rutin, as well as derivatives from these compound classes to be compared. Although these compound classes are ubiquitous within the *Rutaceae* and the plant kingdom, the coumarin geiparvarin **3** has not been reported outside of the *Geijera* genus and it would be useful to conduct further research to establish if this compound can be hypothesised as being a potential chemotaxonomic marker. It was noted that the most abundant compounds present in the various plant part extracts differed only in their quantities, with no additional metabolites observed for the different plant parts studied. This is evident in the analytical HPLC chromatograms for the plant parts of the crude extracts when analysed and assessed at the two wavelengths of 220 nm and 332 nm, respectively (see Figure 2 and Figure 3). The bark extracts contained a larger variety of alkaloid and coumarin derivatives than the extracts from the leaves, fruits, and flowers. In contrast, the flower extracts were composed almost entirely of large proportions of the dominant compounds, with few other minor constituents present. This was observed in the HPLC chromatograms of both the DCM and MeOH crude extracts. Geiparvarin **3** and N-(acetoxymethyl) flindersine **2** were the two most dominant metabolites in all extracts, as evidenced by the largest peaks observed in the chromatograms.

Recent research on the genus *Geijera* is limited due to the small number of species that it contains as well as their remote geographical occurrence. However, recent studies on alkaloids and coumarins from other members of the *Rutaceae* have been promising. For example, auraptene **9** isolated from *Clausena excavata* has displayed some potential as an antidiabetic, with an increase in glucose consumption in 3T3-L1 adipocyte cells by 54.67% as well as moderate glucose uptake with a ratios 1.38-fold compared to the positive control (metformin, 2.25-fold) [26]. The antinociceptive, anti-inflammatory and antioxidant properties of constituent alkaloids and coumarins which are in keeping with the traditional use of *Fagaropsis hildebrandtii* in Kenya, were corroborated through various assays [27]. Alkaloids and coumarins from *Clausena lansium* which is used traditionally in China and Southeast Asia to treat bronchitis, asthma, hepatitis, and gastrointestinal disorders have displayed significant anti-inflammatory properties [28,29]. *Evodia lepta,* a herb used in traditional Chinese medicine (TCM) to treat chronic inflammatory conditions such as arthritis as well as infections such as influenza, was found to contain a racemic mixture of an alkaloid with significant anti-neuroinflammatory activity, and the use of other compounds from this plant have been suggested for the treatment of dementia [30,31]. Assays of constituent compounds from the bark of *Zanthoxylum gilletii* revealed significant activity again *Plasmodium falciparum*, which supports the traditional use of this plant in Kenya and the Ivory Coast to treat malaria [32]. These are some examples, however, there are several studies within recent research, where alkaloids and coumarins as well as extracts containing these compound classes from various other members of the *Rutaceae* have continued to demonstrate new biological activities as well as corroborating traditional activities that have already been established.

A summary of the compounds observed in each of the plant parts for the main chromatographic peaks as obtained via HPLC-DAD and LC-MS is provided in Table 3. Their structures were confirmed via NMR spectroscopy, which corroborates the identification of the dominant compounds, performed based on matching the masses and UV maxima of the compounds. The peaks present in the MeOH crude extracts around the retention time of 10 min (see Table 3) exhibited diagnostic UV maxima typical of *O*-substituted quercetins; in this case, the largest of these peaks represents the glycoside flavonol, rutin (610 amu), which is one of the most well-known and ubiquitous of this compound class.

### 3.4. Anthelmintic Activity Assessment

*G. parviflora* extracts and compounds were evaluated against xL3s of *H. contortus* to establish whether they inhibited larval motility and/or development, and/or induced a non-wildtype morphology (abnormal phenotype). The crude extracts were assayed at a single concentration (0.5 mg/mL) and compounds were assessed in a dose-response assay (50 µM to 0.2 µM). The effect of the crude extracts and the purified compounds on the motility, development, and phenotype of xL3s at 168 h is summarised in Table 4 and Table 5.

**Figure 4:**
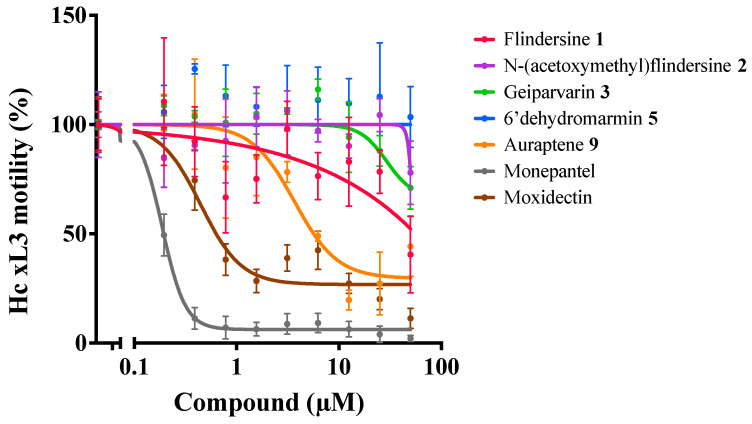
Dose-response curves of *G. parviflora* compounds on the motility of xL3s of *H. contortus* at 168 h.

All the DCM crude extracts were active (≥70% motility reduction) and the resinous components of the MeOH extracts also displayed anthelmintic activity (100% motility reduction) (Table 4). The DCM crude extracts of leaves and flowers and the resinous component of the leaf MeOH extract induced a skinny (*Skn*) phenotype in affected larvae. The solid component of the leaf MeOH extract also displayed some anthelmintic activity, but the MeOH extract of the flowers did not. As a result, the DCM crude extracts of the leaves and bark were prioritised and subjected to further fractionation and compound isolation. Although the compounds from the bark were not obtained in a sufficient quantity for anthelmintic activity assessment, five purified compounds obtained from the leaves were evaluated (Table 5). Of these, auraptene and flindersine 1 exhibited significant activity against *H. contortus* (see Table 5 and Figure 4). Flindersine **1** inhibited motility (IC_50_ 3.7 µM) and auraptene **9** inhibited development (100% at 25 µM) of the xL3 stage of *H. contortus*. In addition, geiparvarin **3** induced a *Skn* phenotype (100% at 21.7 µM) in the affected larvae.

### 3.5. Antimicrobial Activity Assessment

Two coumarins isolated from the bark of *G. parviflora*, namely geiparvarin **3** and 6′-dehydromarmin **5**, were evaluated at a concentration of 32 µg/mL for their antimicrobial activity against seven pathogens including methicillin-resistant *Staphylococcus aureus* (MRSA); four Gram-negative bacteria *Escherichia coli*, *Klebsiella pneumoniae*, *Acinetobacter baumannii*, *Pseudomonas aeruginosa;* and two fungal pathogens *Candida albicans* and *Cryptococcus neoformans var. grubii*. Neither compound displayed any antimicrobial activity.

## 4. Conclusions

In this study, the fruits, flowers, leaves, and bark of *G. parviflora* were chemically profiled and studied. It was concluded that two compounds isolated from the leaves of the plant displayed significant anthelmintic activity.

A comparison of the phytochemical profiles of the various plant parts revealed that the major constituents are present in all parts of the plant, although they vary in the proportions present. The coumarin geiparvarin **3** is the most prevalent in all the parts of this plant. Since this compound is specific to the genus *Geijera*, further research may reveal if it can be hypothesised as being a chemotaxonomic marker for the genus.

This study resulted in the isolation of seven previously described compounds, including auraptene, geiparvarin, flindersine, 6′-dehydromarmin, marmin acetonide, as well as two flindersine derivatives, namely N-(acetoxymethyl) flindersine and 3′,4′- dihydroxy-3′,4′-dihydroflindersine. These are the major compounds that contribute to the biological activities of the plant, and their activities corroborate the use of *G. parviflora* in Australian bush medicine. In addition, a new compound, chlorogeiparvarin **6**, was isolated as a mixture with geiparvarin **3**.

Considering their previously established bioactivities, it was of interest to explore the therapeutic potential of some of these compounds in a different context, unrelated to the customary use of the plant. Several of the crude extracts of the plant, as well as compounds isolated separately from leaves and bark, were subjected to anthelmintic and antimicrobial testing, leading to the discovery that flindersine and auraptene display significant anthelmintic activity against the parasitic nematode *H. contortus*. This is the first reported instance of this anthelmintic activity for *G. parviflora* extracts and for these two phytochemical constituents, for which other activities have been reported—in the case of flindersine, antibacterial, anti-inflammatory, collagen III suppression, and antifungal; and for auraptene, an increase in collagen I expression, antibacterial, antifungal, antileishmanial, antidiabetic, anticancer, neuroprotective, and antioxidant activities.

This study demonstrates the importance of exploring, in detail, the constituents of individual plant parts and in evaluating the bioactivity of pure compounds in a variety of biological assays. This approach has the potential to lead to the discovery of new and yet undescribed phytochemical compounds with therapeutic activities.

## Figures and Tables

**Table 1:** NMR chemical shifts and structure elucidation of compound **6**.

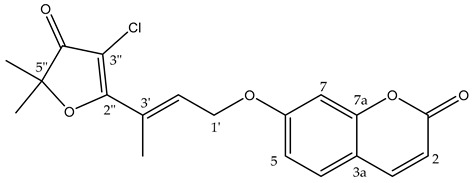				
Position	Carbon, Type	Proton, Multiplicity (J in Hz)	gCOSY	gHMBCAD
1	161.2, C			
2	113.7, CH	6.28, (d, *J* = 9.4 Hz, 1H)	3	1
3	143.4, CH	7.65, (d, *J* = 9.4 Hz, 1H)	2	1, 4, 7a
3a	113.2, C			
4	129.1, CH	7.41, (d, *J* = 8.7 Hz, 1H)	5	6, 7a
5	113.1, CH	6.88, (m, 1H)	4	
6	161.47, CH			
7	101.7, CH	6.83, s		3a, 5
7a	156.0, C			
1′	65.36, CH_2_	4.86, (d, *J* = 5.9 Hz, 2H)	2′, 3′-CH_3_^w^	6, 2′, 3′
2′	135.0, CH	6.95, (t, *J* = 5.9 Hz, 1H)	1′, 3′-CH_3_^w^	
3′	129.16, C			
2″	176.4, C			
3″	ND	ND		
4″	200.6, C			
5″	87.2, C			
3′-CH_3_	14.1, CH_3_	2.15, s	1′ ^w^, 2′ ^w^	2′, 3′, 2″,
5″-CH_3_	23.4, CH_3_	1.45, s		4″, 5″, 5″-CH_3_

Recorded at 500 MHz in CDCl_3_; ^w^ indicates weak or long-range correlation; ND—not detected; Note: Carbons 6, 1′, 3′ are listed to 2 decimal places as they were different from the corresponding carbons at these locations in the NMR of geiparvarin **3**.

**Table 2:** Comparison of the NMR chemical shifts (ppm) of compounds **6** and **3** (isolated as a mixture).

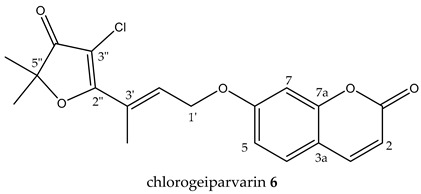			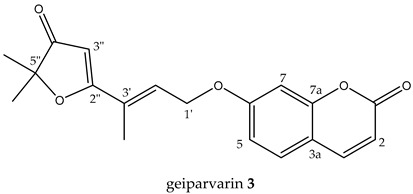	
Position	Carbon, Type	Proton, Multiplicity (*J* in Hz)	Carbon, Type	Proton, Multiplicity (*J* in Hz)
1	161.2, C		161.2, C	
2	113.7, CH	6.28, (d, *J* = 9.4 Hz, 1H)	113.7, CH	6.28, (d, *J* = 9.4 Hz, 1H)
3	143.4, CH	7.65, (d, *J* = 9.4 Hz, 1H)	143.4, CH	7.65, (d, *J* = 9.4 Hz, 1H)
3a	113.2, C		113.2, C	
4	129.1, CH	7.40, (d, *J* = 8.7 Hz, 1H)	129.1, CH	7.40, (d, *J* = 8.7 Hz, 1H)
5	113.1, CH	6.88, (m, 1H)	113.1, CH	6.88, (m, 1H)
6	161.47, CH		161.57, C	
7	101.7, CH	6.83, s	101.7, CH	6.83, s
7a	156.0, C		156.0, C	
1′	65.36, CH_2_	4.86, (d, *J* = 5.9 Hz, 2H)	65.33, CH_2_	4.83, (d, *J* = 5.9 Hz, 2H)
2′	135.0, CH	6.95, (t, *J* = 5.9 Hz, 1H)	130.5, CH	6.75, (t, *J* = 5.9 Hz, 1H)
3′	129.16, C		128.94, C	
2″	176.4, C		183.0, C	
3″	ND		100.5, CH	5.62, s
4″	200.6, C		207.4, C	
5″	87.2, C		88.9, C	
3′-CH_3_	14.1, CH_3_	2.15, s	14.0, CH_3_	2.03, s
5″-CH_3_	23.4, CH_3_	1.45, s	23.2, CH_3_	1.41, s

ND—not detected.

**Table 3:** Dominant constituents in different *G. parviflora* plant parts detected via LC-MS.

Retention Time (min)	UV Maxima (nm)	Negative Ion *m*/*z*	Positive Ion *m*/*z*	Compound Name & Molecular Weight (amu)
10.2	202, 220, 284, 326	609 [M − H]^−^	611 [M + H]^+^	Rutin 610
14.1	204, 222, 324	-	333 [M + H]^+^	Marmin 332
15.4	192, 222, 346	226 [M − H]^−^	228 [M + H]^+^	Flindersine 227
15.8	202, 220, 322	-	331 [M + H]^+^	6′-dehydromarmin 330
16.6	200, 218, 314	325 [M − H]^−^	327 [M + H]^+^	Geiparvarin 326
18.3	224, 350	298 [M − H]^−^	300 [M + H]^+^	N-(acetoxymethyl) flindersine 299
26.0	230, 322	-	299 [M + H]^+^	Auraptene 298

**Table 4:** Anthelmintic activity of *G. parviflora* crude extracts (0.5 mg/mL) against xL3s of *H. contortus* after 168 h.

Description	Motility Reduction	Development Inhibition	Abnormal Phenotype Induction
**Dichloromethane extract of the bark**	≥70%	Nil.	Nil.
**Methanol extract of the bark (resin)**	100%	100%	Nil.
**Methanol extract of the bark (solids)**	Nil.	Nil.	Nil.
**Dichloromethane extract of the leaves**	≥70%	Nil.	*Skn*
**Methanol extract of the leaves (resin)**	100%	Nil.	*Skn*
**Methanol extract of the leaves (solids)**	≥70%	Nil.	Nil.
**Dichloromethane extract of the flowers**	≥70%	Nil.	*Skn*
**Methanol extract of the flowers**	Nil.	Nil.	Nil.

Nil. = no effects; *Skn* = skinny phenotype.

**Table 5:** Anthelmintic activity of *G. parviflora* isolated compounds as evaluated in a dose-response assay against xL3s of *H. contortus* at 168 h.

Description	Motility Reduction (IC_50_; µM)	Development Inhibition	Abnormal Phenotype Induction
**Compound 1** **flindersine**	3.7 µM	Nil.	Nil.
**Compound 2** **N-(acetoxymethyl)flindersine**	>50 µM	Nil.	Nil.
**Compound 3** **geiparvarin**	>50 µM	Nil.	100% *Skn* at 21.7 µM
**Compound 5** **6′dehydromarmin**	>50 µM	Nil.	Nil.
**Compound 9** **auraptene**	>50 µM	100% at 25 µM	Nil.
**Monepantel (control)**	0.2 µM	100% at 0.8 µM	100% *Coi* at 6.3 µM
**Moxidectin (control)**	0.4 µM	100% 25 µM	Nil.

Nil. = no effects; *Skn* = skinny phenotype; *Coi* = Coiled phenotype. The purified compounds listed above were isolated from dichloromethane extract of *G. parviflora* leaves.

## Data Availability

The original contributions presented in the study are included in the article/Supplementary Material, further inquiries can be directed to the corresponding author/s.

## Supplementary Materials

The following supporting information can be downloaded at: https://www.mdpi.com/article/10.3390/metabo14050259/s1, Scheme S1.1: Extraction and isolation of compounds from the bark of *G. parviflora*; Scheme S2.1: Extraction and isolation from the leaves of *G. parviflora*; Figure S3.1: ^1^H NMR Spectrum of compound **6** (500 MHz, CDCl_3_), Figure S3.2: gCOSY NMR Spectrum of compound **6** chlorogeiparvarin (500 MHz, CDCl_3_), Figure S3.3: HSQCAD NMR Spectrum of compound **6** chlorogeiparvarin (500 MHz, CDCl_3_), Figure S3.4: HMBCAD NMR Spectrum of compound **6** chlorogeiparvarin (500 MHz, CDCl_3_).
