# Supplementary material for: Phytochemical Profiling Studies of Alkaloids and Coumarins from the Australian Plant Geijera parviflora Lindl. (Rutaceae) and Their Anthelmintic and Antimicrobial Assessment
Source: Metabolites. 2024 Apr 30;14(5):259. doi: 10.3390/metabo14050259 (PMC11123165; doi:10.3390/metabo14050259)
Supplement: Supplementary file 1 [file metabolites-14-00259-s001.zip › metabolites-2974970-supplementary.pdf]

A phytochemical and bioactivity study of the Australian plant *Geijera parviflora*

Deepika Dugan [1], Rachael J. Bell [1], Robert Brkljača [2], Colin Rix [1], Aya C. Taki [3], Robin B. Gasser [3] and Sylvia Urban [1,*]

[1] School of Science (Applied Chemistry and Environmental Science), Marine and Terrestrial Natural Product (MATNAP) Research Group, RMIT University, Melbourne, VIC 3001, Australia; s3646443@student.rmit.edu.au (D.D.); rbell0030@gmail.com (R.J.B.); colin.rix@rmit.edu.au (C.R.)

[2] Monash Biomedical Imaging, Monash University, Clayton, VIC 3168, Australia; robert.brkljaca@monash.edu (R.B.)

[3] Department of Veterinary Biosciences, Melbourne Veterinary School, Faculty of Science, The University of Melbourne, Parkville, VIC 3010, Australia; aya.taki@unimelb.edu.au (A.C.T.); robinbg@unimelb.edu.au (R.B.G.)

* Correspondence: sylvia.urban@rmit.edu.au

Supporting Information

S1

Scheme S1.1 Extraction and isolation of compounds from the bark of *G. parviflora*

S2

Scheme S2.1 Extraction and isolation from the leaves of *G. parviflora*

S3

Figure S3.1 ^{1}H NMR Spectrum of compound **6** chlorogeiparvarin (500 MHz, $CDCl_3$)

Figure S3.2 gCOSY NMR Spectrum of compound **6** chlorogeiparvarin (500 MHz, $CDCl_3$)

Figure S3.3 HSQCAD NMR Spectrum of compound **6** chlorogeiparvarin (500 MHz, $CDCl_3$)

Figure S3.4 HMBCAD NMR Spectrum of compound **6** chlorogeiparvarin (500 MHz, $CDCl_3$)

S1 **Scheme S1.1** Extraction and isolation of compounds from the bark of *G. parviflora*

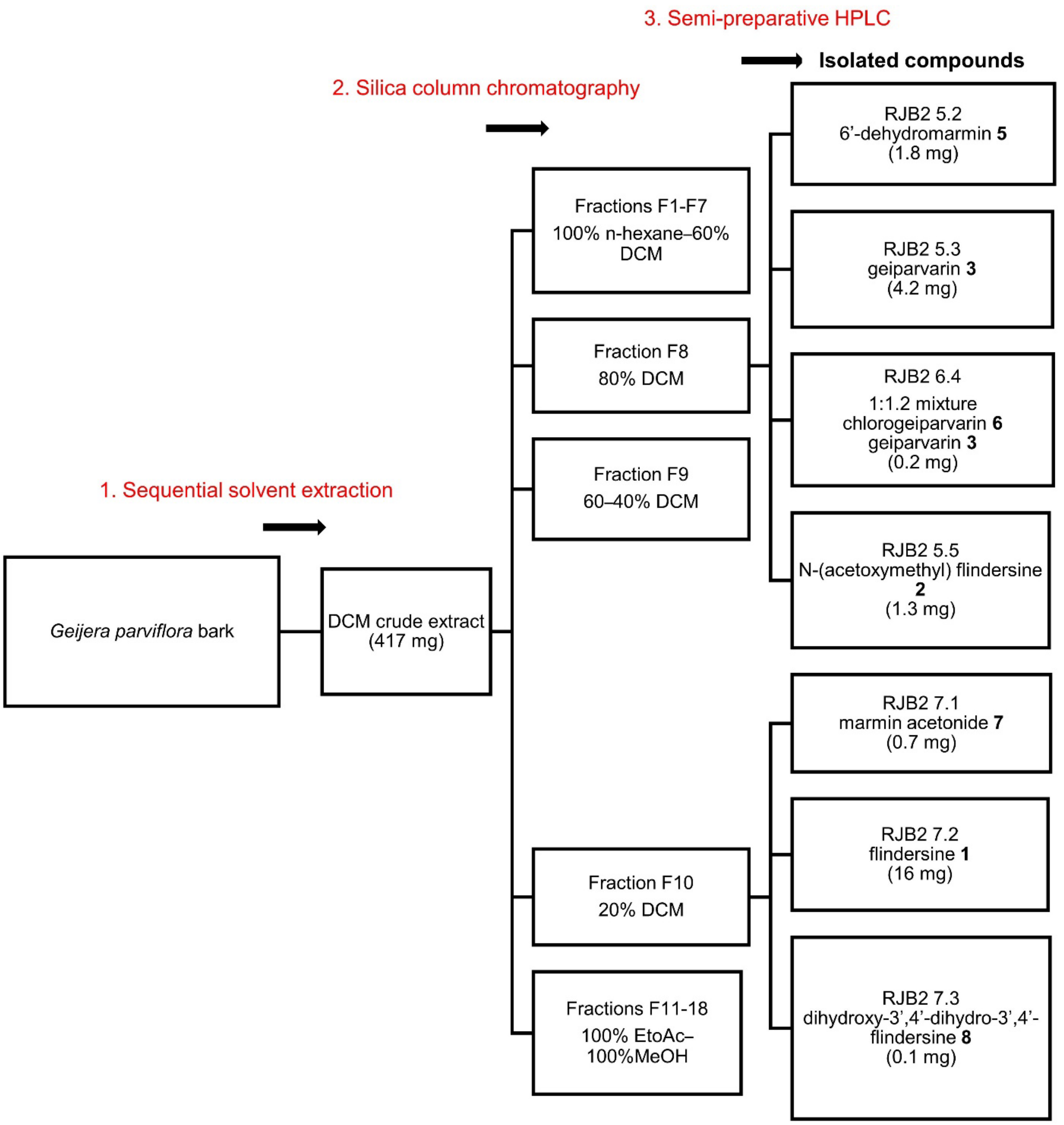

1. Sequential solvent extraction of the bark of *Geijera parviflora* was undertaken using DCM followed by MeOH. This employed a solvent trituration methodology to exhaustively extract the bark with DCM followed by an exhaustive extraction with MeOH respectively.
2. Silica flash column chromatography was performed using ~ 40–50 g Merck Silica gel 60 (0.040–0.063 mm) with a 1:10 loading capacity. A 20% stepwise solvent elution gradient was employed for fractionation of the crude DCM extracts of the bark using increasingly polar solvents commencing with-100% n-hexane to 100% DCM to 100% EtOAc and finally to 100% MeOH respectively.
3. Reversed phase semi-preparative HPLC was carried out on fractions F8 and F10 employing 50 µL injections on a Varian Prostar 210 solvent delivery system equipped with a 250 x 9.4 mm, Agilent Eclipse XDB C18 (5 µm) column and a Prostar 335 PDA detector operated using "Star Workstation" software;

monitoring at wavelengths of 210 nm and 330 nm. Isocratic HPLC methods were used with a solvent ratio of 55% CH_3CN: 45% H_2O and a flow rate of 3.5 mL/min.

S2 **Scheme S2.1** Extraction and isolation from the leaves of *G. parviflora*

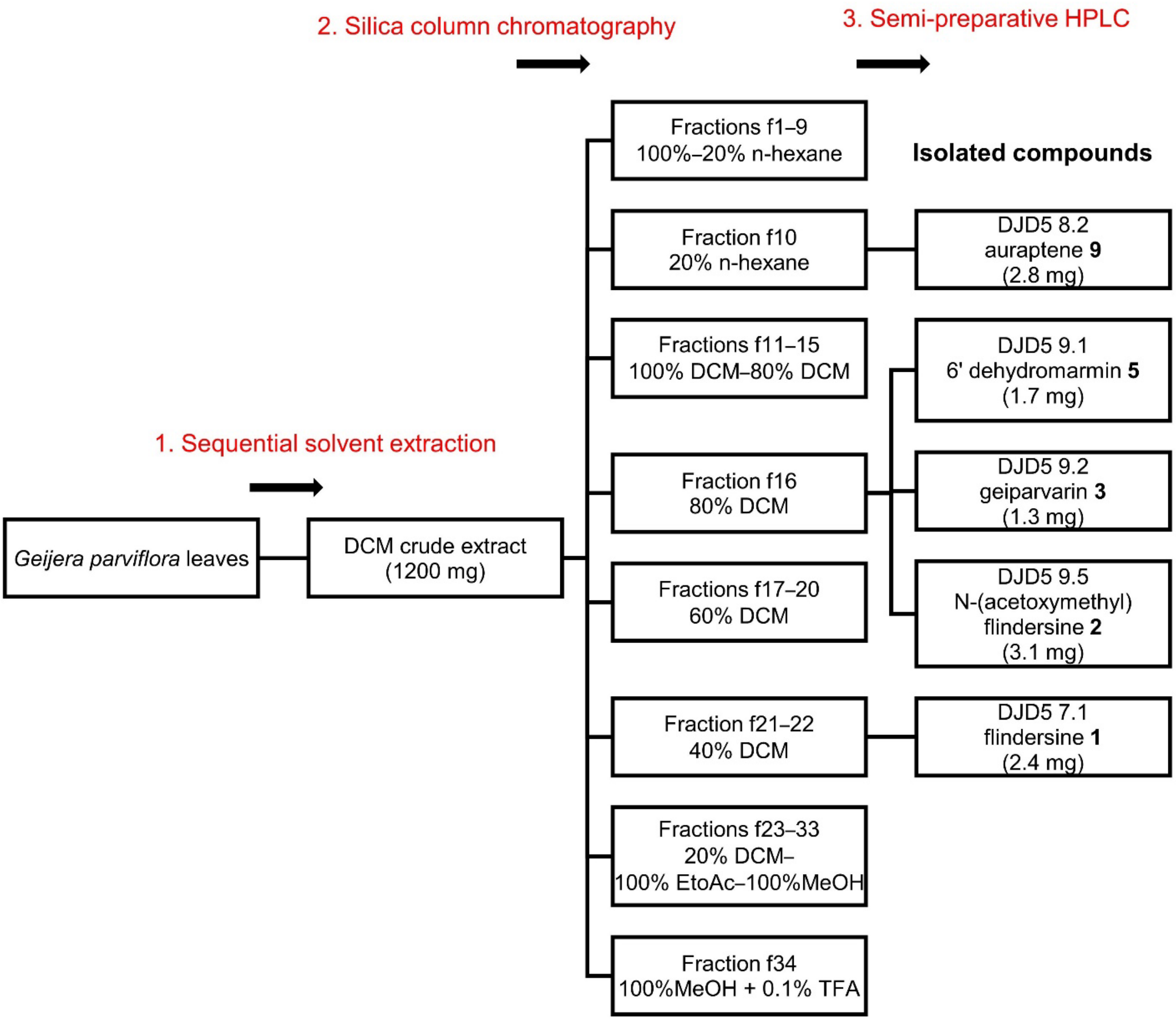

1. Sequential solvent extraction of the leaves of *Geijera parviflora* was undertaken using DCM followed by MeOH. This employed a solvent trituration methodology to exhaustively extract the leaves with DCM followed by an exhaustive extraction with MeOH respectively.
2. Silica flash column chromatography was performed using ~ 40–50 g Merck Silica gel 60 (0.040–0.063 mm) with a 1:10 loading capacity. A 20% stepwise solvent elution gradient was employed for fractionation of the crude DCM extracts of the leaves using increasingly polar solvents commencing with- 100% n-hexane to 100% DCM to 100% EtOAc and finally to 100% MeOH respectively.
3. Reversed phase semi-preparative HPLC was carried out on selected fractions employing 30 μL injections on a Varian Prostar 210 solvent delivery system equipped with a 250 x 9.4 mm, Agilent Eclipse XDB C18 (5 μm) column and a Prostar 335 PDA detector operated using "Star Workstation" software; monitoring at wavelengths of 210 nm and 330 nm. Isocratic HPLC methods were used with solvent ratio of 80% CH_3CN: 20% H_2O at a flow rate of 4 mL/min for fraction f10, 60% CH_3CN: 40% H_2O at a flow rate of 3.5 mL/min for fraction f16, 50% CH_3CN: 50% H_2O a flow rate of 3.5 mL/min for fraction f21.

S3

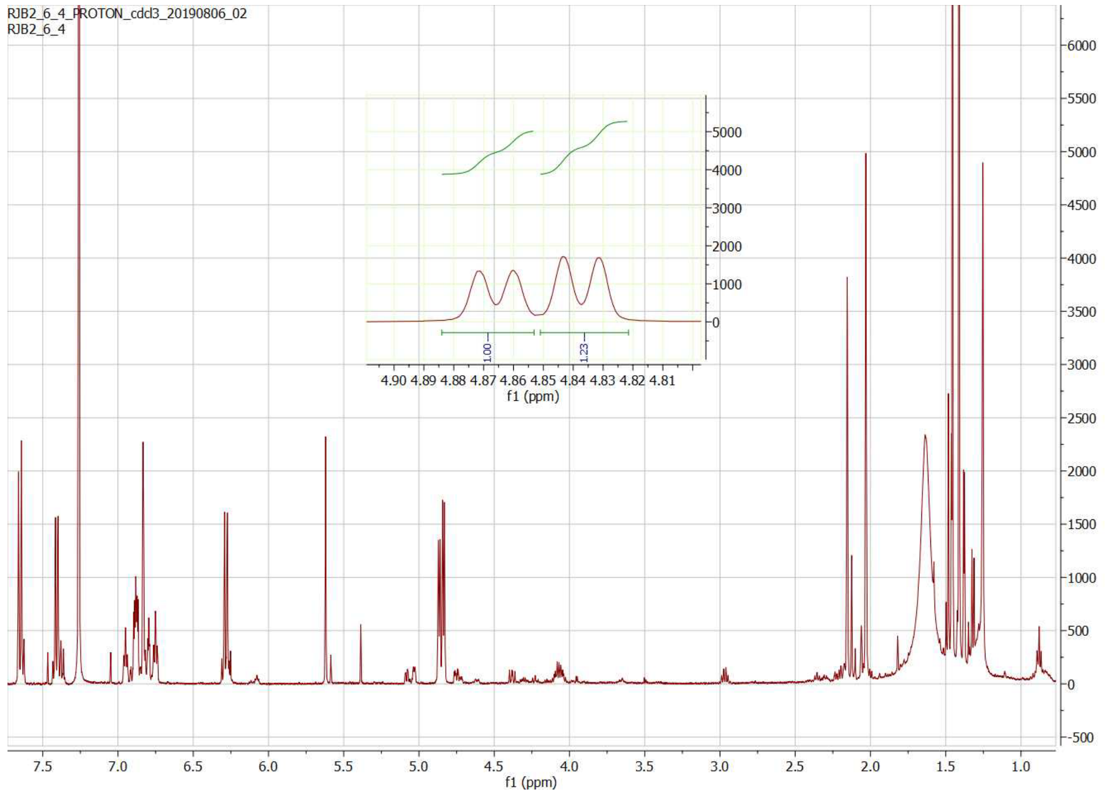

Figure S3.1 ^{1}H NMR Spectrum of compound **6** chlorogeiparvarin (500 MHz, $CDCl_3$)

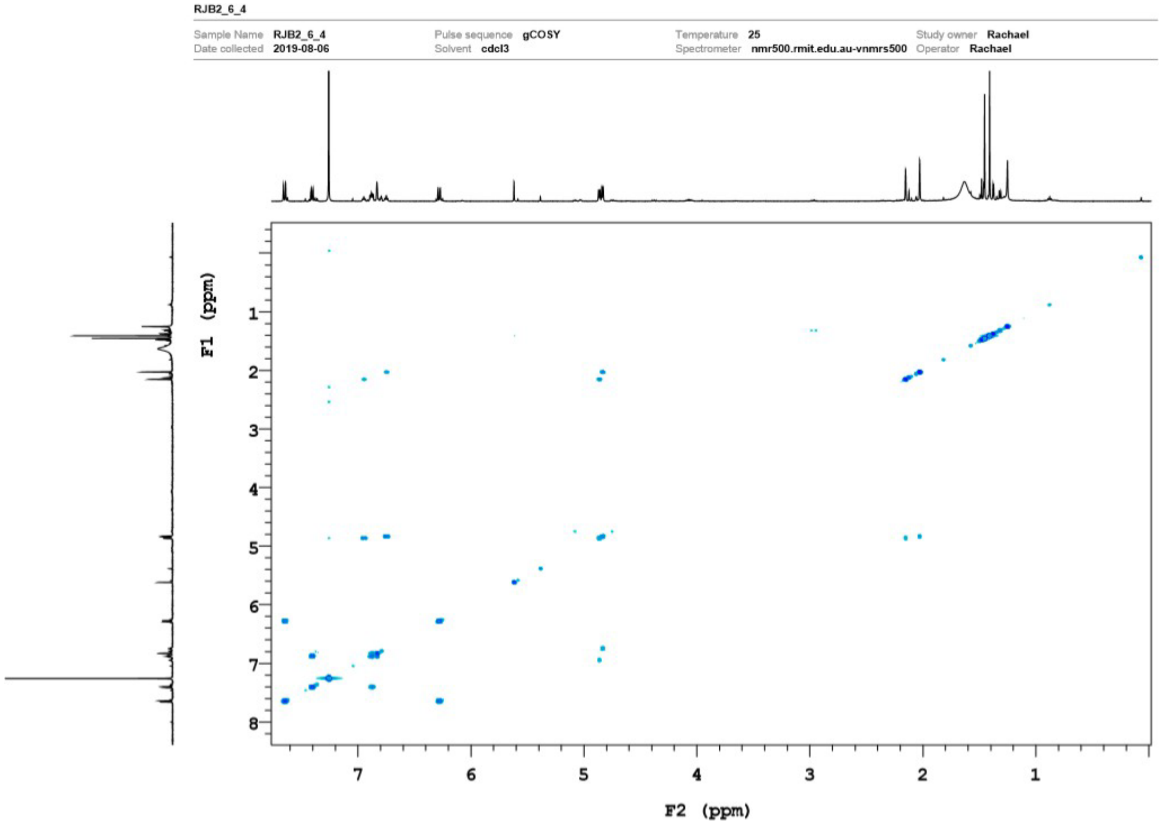

Figure S3.2 gCOSY NMR Spectrum of compound **6** chlorogeiparvarin (500 MHz, $CDCl_3$)

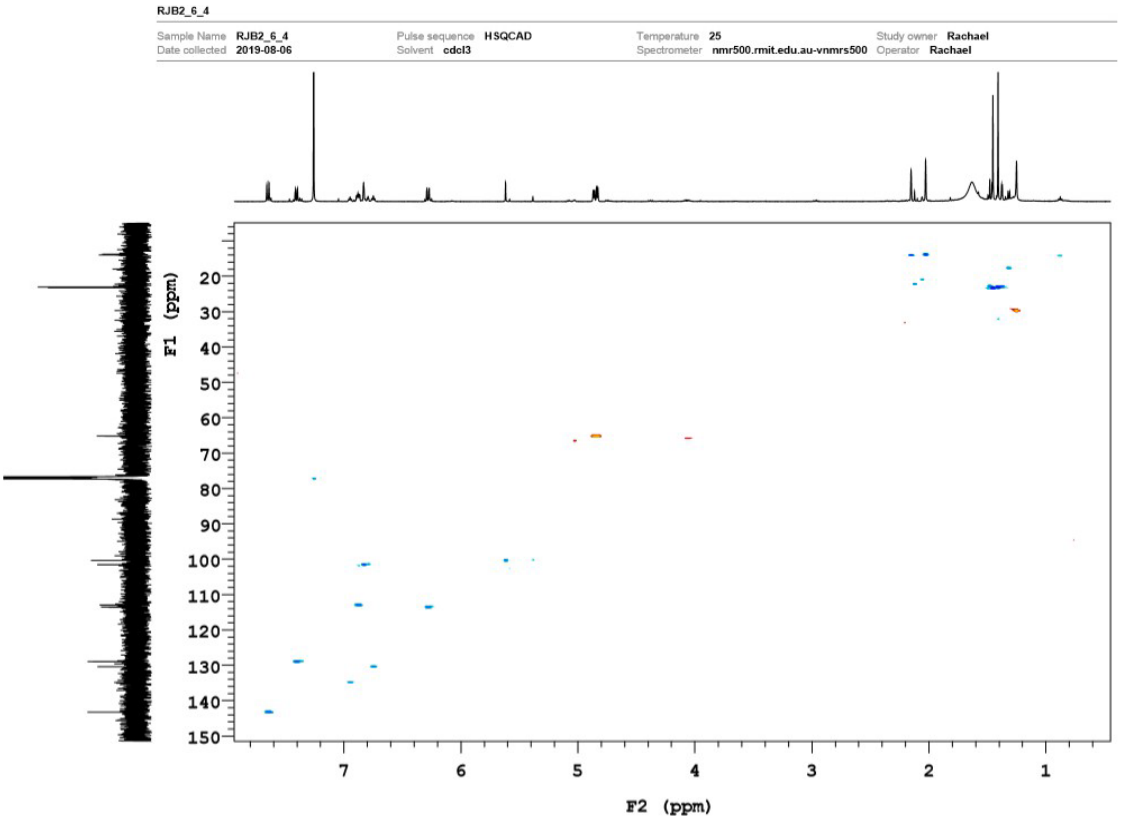

Figure S3.3 HSQCAD NMR Spectrum of compound **6** chlorogeiparvarin (500 MHz, $CDCl_3$)

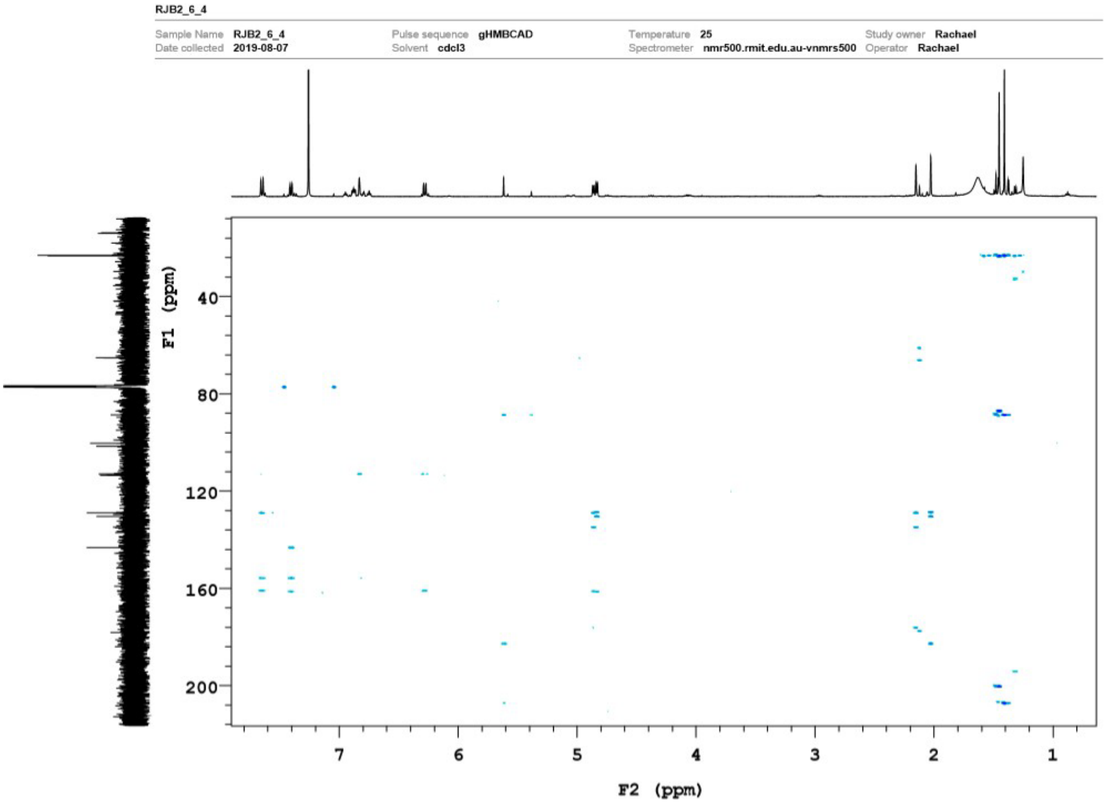

Figure S3.4 HMBCAD NMR Spectrum of compound **6** chlorogeiparvarin (500 MHz, $CDCl_3$)
